# *Mentha suaveolens* Ehrh. (Lamiaceae) Essential Oil and Its Main Constituent Piperitenone Oxide: Biological Activities and Chemistry †

**DOI:** 10.3390/molecules20058605

**Published:** 2015-05-13

**Authors:** Mijat Božović, Adele Pirolli, Rino Ragno

**Affiliations:** Rome Center for Molecular Design, Department of Drug Chemistry and Technology, Sapienza University, P.le Aldo Moro 5, 00185 Rome, Italy; E-Mails: mijatboz@gmail.com (M.B.); adele.pirolli@uniroma1.it (A.P.)

**Keywords:** *Mentha suaveolens* Ehrh., essential oil, extracts, piperitenone oxide, biological activity

## Abstract

Since herbal medicines play an important role in the treatment of a wide range of diseases, there is a growing need for their quality control and standardization. *Mentha suaveolens* Ehrh. (MS) is an aromatic herb with fruit and a spearmint flavor, used in the Mediterranean areas as a traditional medicine. It has an extensive range of biological activities, including cytotoxic, antimicrobial, antioxidant, anti-inflammatory, hypotensive and insecticidal properties, among others. This study aims to review the scientific findings and research reported to date on MS that prove many of the remarkable various biological actions, effects and some uses of this species as a source of bioactive natural compounds. On the other hand, piperitenone oxide (PO), the major chemical constituent of the carvone pathway MS essential oil, has been reported to exhibit numerous bioactivities in cells and animals. Thus, this integrated overview also surveys and interprets the present knowledge of chemistry and analysis of this oxygenated monoterpene, as well as its beneficial bioactivities. Areas for future research are suggested.

## 1. Introduction

Essential oils are volatile, natural, complex compound mixtures characterized by a strong odor. They arise from the secondary metabolism of the plant, normally formed in special cells or groups of cells or in glandular hairs found on many leaves and stems. Essential oils are variable mixtures composed principally of terpenoids, including monoterpenes and sesquiterpenes (diterpenes may also be present), and their oxygenated derivatives. A variety of other molecules may also occur, such as aliphatic hydrocarbons, acids, alcohols, aldehydes, acyclic esters or lactones, and exceptionally nitrogen- and sulphur-containing compounds, coumarins and phenylpropanoid homologues. Known for their antiseptic (*i.e.*, bactericidal, virucidal and fungicidal), medicinal properties and their fragrance, they are used in embalmment, preservation of foods and as antimicrobial, analgesic, sedative, anti-inflammatory, spasmolytic and local anesthetic remedies [1].

The spread of drug-resistant pathogens is nowadays one of the most serious threats to the successful treatment of microbial diseases. It has been well-known since ancient times that certain plants and spices have antimicrobial activity [2,3]. They produce an enormous array of secondary metabolites, and it is commonly accepted that a significant part of this chemical diversity serves to protect plants against microbial pathogens. The World Health Organization (WHO) has noted that a majority of the World’s population depends on traditional medicine for its primary healthcare [4].

**Figure 1:**
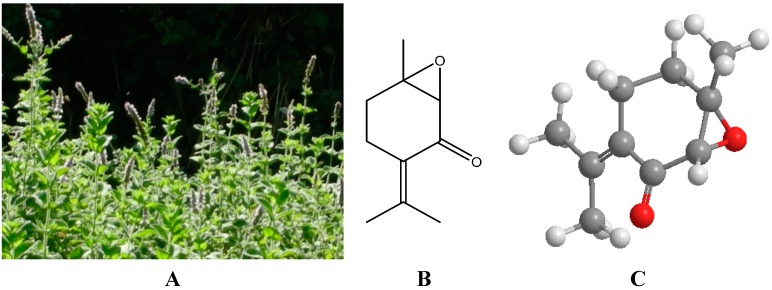
Wild MS (**A**) and PO 2D (**B**) and 3D (**C**) structure depictions.

Many essential oils and their ingredients have been shown to exhibit a range of biological activities, including antibacterial and antifungal activity. As a result, essential oils and/or their components are becoming increasingly popular as natural antimicrobial agents used for a wide variety of purposes. Their preparations find applications as naturally occurring antimicrobial agents in pharmacology, pharmaceutical botany, phytopathology, medical and clinical microbiology and food preservation. This review focuses on the essential oil of *Mentha suaveolens* Ehrh. (EOMS) and one of its main chemical constituents—piperitenone oxide (PO) (Figure 1).

## 2. *Mentha*: Species, Taxonomy, Occurrence and Uses

Based on phylogenetic analysis of morphology, chromosome numbers and major essential oil constituents the genus *Mentha* (mint), an important member of the *Lamiaceae* family, is highly diverse [5,6]. It is represented by about 19 species and 13 natural hybrids, mainly perennial herbs, growing wildly in damp or wet places throughout the temperate regions of Europe, Asia, Africa, Australia and North America. Mints are fast growing, invasive and generally tolerate a wide range of agro-climatic conditions [7].

**Scheme 1:**
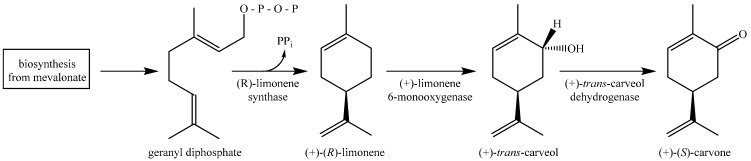
Carvone biosynthetic pathway.

*Mentha* identification is difficult, since in addition to much phenotypic plasticity and genetic variability, most of the species are capable of hybridization with each other. Hybrids are frequent in Nature but can usually be recognized by their intermediate appearance and sterility, although fertile hybrid swarms occasionally occur [8]. The present literature suggests the classification of the genus *Mentha* into the three basic lines named: *capitatae*, *spicatae* and *verticillatae*, based on the characteristic inflorescence. The *capitatae* line includes all species with compact, head-like inflorescences; the type species is *M. aquatica*. The *spicatae* species have a spike, as shown by *M. spicata*, *M. longifolia* and MS. The third line, represented by *M. arvensis*, has an inflorescence vertically partitioned into whorls [9]. Mints are also classified based on the dominant monoterpene compound prevailing in the essential oil resulting from three different metabolic pathways. Thus, the production of linalool and linalyl acetate is typical for the linalool pathway; menthol, menthone and menthofuran are constituents of the menthol pathway, and carvone, dihydrocarvone and carveol characterize the carvone pathway (Scheme 1) [5,9,10].

Eight species are reported in the Italian flora: *M. requienii*, *M. pulegium*, *M. arvensis* (and hybrids), *M. aquatica* (and hybrids) and the *M. spicata* group which includes *M. spicata*, *M. longifolia*, *M. microphylla* and MS [11]. *Mentha* species have been known for their medicinal and aromatherapeutic properties since ancient times. The ancient Egyptians, Romans and Greeks used peppermint as a flavoring agent for food and as a medicine, while mint essential oils have been used as perfumes, food flavors, deodorants and pharmaceuticals [7]. During the Middle Ages, powdered mint leaves were used to whiten the teeth [12]. Leaves, flowers and stems of *Mentha* spp. are frequently used in herbal teas or as additives in commercial spice mixtures for many foods to offer aroma and flavor. In addition, mints have been used as a folk remedy for treatment of nausea, bronchitis, flatulence, anorexia, ulcerative colitis and liver complaints, due to their anti-inflammatory, carminative, antiemetic, diaphoretic, antispasmodic, analgesic, stimulant, emmenagogue and anticatharrhal activities [13,14,15,16]. Different mint species are also used for rheumatism, dysentery, dyspepsia, skin allergies, chills, jaundice, throat infections, constipation, spasms, bladder stones, gall stone, diarrhea, toothache, stomach aches, dyspnea, gastrodynia, and as stimulant, diaphoretic, diuretic, reconstituent, stomach tonic, anti-infective, sedative, insect repellent, antimycobacterial, antifungal, antiallergic, virucidal, radioprotective, cyclooxygenase inhibitor, anti-inflammatory and hemostatic agents [17,18].

## 3. *Mentha* Essential Oils

Recently, essential oils and various extracts of plants have provoked interest as sources of natural products. They have been screened for their potential uses as alternative remedies for the treatment of many infectious diseases and the preservation food from the toxic effects of oxidants. Research on plants from different regions has led to innovative ways to use the essential oils [19]. Particularly, the antimicrobial activities of plant oils and extracts have formed the basis of many applications, including raw and processed food preservation, pharmaceuticals, alternative medicine and natural therapies [15].

Members of the genus *Mentha* produce some of the most widely used essential oils. Different species vary in their essential oil content and composition. The biosynthesis and metabolism of essential oils are strongly influenced by environmental factors, such as temperature, photoperiod, nutrition and salinity [20]. Plant chemotypes, cultivation practices and method of extraction also lead to variations in oil content and composition. Other factors affecting essential oil composition relate to agronomic and genotype conditions, such as harvesting time, plant age and crop density [7].

The essential oil isolated from mint leaves has economic importance and is widely used in the food industry, cosmetics, confectionary and pharmaceutical industries [5,21] Mint is widely cultivated for this oil, produced in many countries, such as America, India, China and Canada [5]. Commercially, the most important mint species are peppermint (*M. x piperita*), spearmint (*M. spicata*) and corn (American wild) mint (*M. canadensis*). Many species have been studied experimentally and the efficiency of some traditional applications were confirmed in many reports.

## 4. *Mentha suaveolens* Ehrh.: Taxonomic Characterization, Distribution and Uses

MS, apple mint, woolly mint or round-leafed mint (synonyms: *M. macrostachya* Ten., *M. insularis* Req.) is an herbaceous, perennial herb with a sickly sweet scent that grows up to 100 cm in height (Figure 1). The stem is erect, quadrangular, and sparsely hairy to densely white-tomentose. It is monopodially branched, with short internodes. The foliage is light green with opposite, wrinkled, sessile or very short petiolate leaves which are ovate-oblong to suborbicular, 3 to 4.5 cm long and 2 to 4 cm broad. Obtuse, cuspidate or rarely acute, the leaves are widest near the base, serrate, with 10–20 teeth, hairy above, usually grey or white-tomentose to lanate. A prostrate branch (creeping sucker) growing from the axil of the leaves at the base of the flowering stem propagates below the level of the ground then gives root and turns upwards to give a new shoot. Many verticillasters, usually congested, form a terminal spike 4 to 9 cm long, consisting of a number of white or pinkish flowers [8,11,22].

This species is native to Southern and Western Europe, extending northwards to The Netherlands, cultivated as a pot-herb and naturalized in Northern and Central parts of Europe. It is generally found along streams, bogs and humid places [23]. MS has been used in the traditional medicine of Mediterranean areas and has a wide range of effects: hypotensive, stimulating, stomachic, carminative, choleretic, antispasmodic, sedative, tonic, anti-convulsive, insecticidal, *etc*. It is also useful in cases of cough, nausea, anorexia and bronchitis [22] and finds application in digestion problems, influenza, respiratory ailments, rheumatism, skin diseases and irritation [24]. It shows depressor, analgesic, anti-inflammatory, cytotoxic, hepatoprotective and antifungal activities [10,14,25]. On reviewing the current literature on the phytochemistry of MS, flavonoids were the major constituents isolated from this species [10,26]. Concerning the biological activities, it was found that MS has antihypertensive [27], antioxidant and acetylcholinesterase inhibitory activities [28] and monoamine oxidase inhibitory activity [29]. The essential oil of MS was also found to have candidacidal activity [30,31] and a significant virucidal activity [32].

## 5. Essential Oil Composition of MS

There is an ongoing effort to screen plants used therapeutically in different regions of the World. However, it is well-known that the same taxon growing in different areas may have widely differing chemical components and hence differing biological properties [23]. Ingredients of EOMS have been subjected to a number of studies which have shown a difference in its constituents depending on the region of origin [33,34,35,36,37]. In general, investigations on the chemical composition of the essential oil from different populations collected in various regions showed high percentages of oxides. These include piperitone oxide and piperitenone oxide (PO) as major components [5,10,23,37]. Other chemotypes of this species showed high percentage of alcohols such as menthol [10] or ketones such as pulegone, piperitenone and dihydrocarvone (Table 1) [23,37]. Accordingly, three profiles of EOMS have been defined previously: the first profile is rich in pulegone, the second in PO and the third one contains similar quantities of PO and piperitone oxide [23].

**Table 1:** Chemical structures, names, MWs and CAS numbers of some of the most common constituents in EOMS.

Chemical Structure	Chemical Name	MW	CAS Number
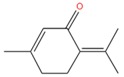	piperitenone	150.22	491-09-8
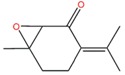	piperitenone oxide	166.22	35178-55-3
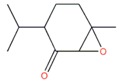	piperitone oxide	168.23	5286-38-4
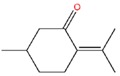	pulegone	152.23	15923-80-6
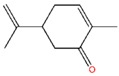	carvone	150.22	6485-40-1

The populations of MS collected in various regions of Morocco showed a high percentage of oxides (PO and piperitone oxide), terpenic alcohols (fenchol, *p*-cymen-8-ol, geraniol, terpineol and borneol) and terpenic ketones (pulegone and piperitenone) [23]. Another investigation on Moroccan plant material from Azrou, Tetouan and Meknès confirmed the prevalence of PO (74.69%, 41.84% and 34%, respectively), while that from M’rirt is rich both in PO (81.67%) and piperitenone (10.14%) (Table 2) [38,39].

**Table 2:** Example of EOMS chemical composition from two Moroccan localities [38].

N	Identified Compound	Kovàts Index KI	Area%	
			Azrou	M’rirt
1	α-Pinene	939	0.36	0.36
2	Camphene	954	-	0.03
3	β-Pinene	979	0.65	0.65
4	*meta*-Mentha-1(7),8-diene	1000	0.18	0.02
5	α-Terpinene	1017	0.07	-
6	*p*-Cymene	1024	0.13	-
7	Limonene	1029	1.85	0.56
8	γ-Terpinene	1059	0.13	-
9	*cis*-Sabinene hydrate	1070	0.53	0.06
10	*trans*-Sabinene hydrate	1098	0.06	-
11	1-Octen-3-yl-acetate	1112	0.13	0.16
12	Dehydrosabinaketone	1120	0.05	-
13	4-Acetyl-1-methylcyclohexene	1137	0.08	-
14	Nopinone	1140	0.05	0.05
15	Borneol	1169	0.27	0.29
16	Terpinen-4-ol	1177	0.71	0.52
17	*p*-Cymen-8-ol	1182	0.12	-
18	α-Terpineol	1188	0.25	0.34
19	Coahuilensol methylether	1221	0.14	-
20	Pulegone	1237	2.34	0.47
21	cis-Carvone oxide	1263	0.44	0.19
22	Geranial	1267	-	0.2
23	Perillaaldehyde	1271	0.17	0.04
24	Isobornylacetate	1285	-	0.1
25	3'-Methoxyacetophenonone	1298	-	0.14
26	Piperitenone	1343	1.17	10.14
27	Piperitenone oxide	1368	74.69	81.67
28	Daucene	1381	0.11	-
29	β-Elemene	1390	0.16	-
30	4a-α,7-β,7a-α-Nepetalactone	1392	1.81	0.42
31	Longifolene	1407	0.27	-
32	β-Caryophyllene	1419	1.68	0.91
33	cis-Muurola-3,5-diene	1450	0.09	-
34	Spirolepechinene	1451	0.16	0.09
35	Khusimene	1455	0.68	-
36	cis-cadina-1(6),4-diene	1463	0.81	0.29
37	γ-Muurolene	1479	5.53	0.5
38	γ-Amorphene	1495	0.30	0.04
39	Aciphyllene	1501	0.10	-
40	γ-Cadinene	1513	0.11	0.1
41	*trans*-Calamenene	1522	0.77	-
42	α-Cadinene	1538	0.09	0.05
43	Spathulenol	1578	0.60	0.25
44	Caryophyllene oxide	1582	0.26	0.21
45	Globulol	1590	0.23	0.06
46	Ledol	1602	-	0.23
47	1,10-di-epi-Cubenol	1619	0.43	0.25
48	10-*epi*-α-Cadinol	1640	0.28	0.04
49	Torreyol	1646	0.05	-
50	α-Cadinol	1654	0.35	0.09
51	Germacra-4(15),5,10(14)trien-1-α-ol	1686	0.07	-
52	Shyobunol	1689	0.10	-

On the other hand, the composition of the essential oils from Béni-Mellal and Boulmane is totally different, with pulegone (85.5%) and menthol (40.50%) as the major compounds, as well as oil from the Oulmès region (Rabat) where piperitenone and pulegone are the main compounds (33.03% and 17.61%, respectively) [38,40]. Dominance of menthol (48.32%) and pulegone (20.27%) is also the characteristic of the material from Córdoba, Argentina [41]. The authors explained that this oil composition was unusual and closely related to that of *Mentha arvensis* var. *piperascens* which raises the possibility that the studied sample was not really MS but rather a hybrid (*M. arvensis* var. *piperiscens* x *M. suaveolens*) [41]. The oil obtained from aerial parts of the wild MS ssp. *timija*, an endemic species of Morocco, was characterised by a very high content of oxygen- containing monoterpenes with menthone (39.4%–10.8%), pulegone (62.3%–34.3%) and isomenthone (9.3%–7.8%) as the main constituents [42].

Hydrodistilled oils of the fresh aerial parts of MS cultivated in Egypt were characterized by carvone and limonene as the major constituents [10,43], while the cultivated material from Beheira (Egypt) showed dominance of linalool (35.32%), *p*-menth-1-en-8-ol (11.08%) and geranyl acetate (10.86%) [20,38]. Wild material analysis from the Alexandria-Cairo desert road (Egypt) showed PO (35.14%), germacrene-D (22.65%), *o*-menth-8-ene (8.98%), *trans*-β-farnesene (6.92%), veridiflorol (7.67%) and l-limonene (5.89%) as the main constituents [6].

MS is also widespread in Corsica, where it is represented by two subspecies: *suaveolens* and *insularis*. The latter species is endemic to the occidental Mediterranean Sea islands of Corsica, Sardinia, and the Balearic Islands. These two subspecies are botanically close, but various morphological characteristics allow their differentiation. Analysis of 59 oil samples isolated from MS wild plants growing in Corsica, followed by statistical analysis of the data, allowed a clear differentiation of both subspecies with respect to the composition of their essential oils: the subspecies *suaveolens* yielded oils dominated either by piperitenone (73.5%) or PO (72%), while all the samples of the *insularis* subspecies contained pulegone and *cis*-*cis*-*p*-menthenolide as main components [37]. Similarly, plant material of *insularis* subspecies from Sardinia is also characterized by prevalence of pulegone [44]. Samples originating from Uruguay and Greece have shown a preponderance of PO that reached 62.4% and 80.8% [33]. A predominance of PO (55%) was also seen in materials from the Czech Republic [45]. However, the same species in northern Algeria contained three different chemotypes: a first one characterized by the predominance of PO (29.36%) and piperitone oxide (19.72%); the second one, conversely, with piperitone oxide (31.4%) as the most abundant component, followed by PO (27.79%), and the third contains piperitenone as major constituent (54.91%) [38]. A prevalence of piperitone oxide (40.5%) followed by hydroxy-*p*-menth-3-one (23.9%) was seen in a sample of cultivated MS in Padua (Italy) [46].

Analysis of the essential oil obtained from wild-type plants grown in the Tarquinia forests (Viterbo, Italy) showed a predominance of PO (>90%), with limonene and 1,8-cineole among minor constituents [25,30,32]. Another study on the same material was performed with the aim to analyze in details the essential oil extraction procedure in term of optimal period and extraction time. The material was submitted to hydrodistillation and the oil was collected at different times (1, 2, 3, 6, 12 and 24 h) on three different days of different months (July, August, September). The amount of the oil varied in function by both day of extraction and separation intervals. In August and September the oil yields were more than 2.5 times that of July. The maximum quantities of the oil are obtained in the first three and during the last twelve hours. In July, in the first 3 h almost 70% of the oil was extracted, while in August and September only 54% and 51%, respectively. In general, the most abundant constituent was PO, which percentage was maximum during the first three hours, disappearing in the last three extractions (after 6, 12 and 24 h). From the point of view of the extraction period, the extracted amounts were higher in the July and August samples than in the September sample. Other constituents are characteristic of the period and become important only after the fourth daily fraction. For example, in the month of July, demelverine and eucalyptol had a percentage of about 0.5%–2.0% in the first 3 h of extraction and increased to reach a maximum of about 29% after the 12-hour extraction. In August, β-caryophyllene oxide was always present and increased from about 0.4% after the 1-hour extraction to a maximum of 15.4% after the 12-hour extraction; cinerolone, that was absent in the first 3 h of extraction, showed high percentage in the next 3 h to reach a maximum of about 38.3% in the 24-hour extraction. In the September period, although PO was still the most abundant compound, the other important components seemed randomly distributed: in the first hour, contrarily from the previous period, β-caryophyllene oxide (13.5%) and cinerolone (18%) were at medium-high percentages, but then β-caryophyllene oxide decreased its percentage while cinerolone was absent but appeared again after 5 h till the last extraction (after 24 h), with a percentage varying from 2.4 to 17.5 [47].

## 6. Antimicrobial Activity of MS

Essential oils and extracts have been used for thousands of years in food preservation, pharmaceuticals, alternative medicine and natural therapies [48]. They are potential sources of novel antimicrobial compounds, especially against bacterial pathogens and, in recent years, a large of number of investigations has been performed on their antimicrobial activities. Antimicrobial evaluations of essential oils are generally difficult because of their volatility, insolubility in water and complex chemistry [13,49].

Because of the mode of extraction, mostly by distillation from aromatic plants, essential oils contain a variety of volatile molecules such as terpenes and terpenoids, phenol-derived aromatic compounds and aliphatic components. The antimicrobial activity of essential oils has been extensively studied and demonstrated against a number of microorganisms, usually using direct-contact antimicrobial assays, such as different types of diffusion or dilution methods, as reviewed by many authors [50]. In these tests, essential oils are brought into direct contact with the selected microorganisms. However, due to high hydrophobicity and volatility of the essential oils, the direct-contact assays face many problems. In diffusion assays, the essential oils components are partitioned through the agar according to their affinity with water, and in dilution methods low water solubility has to be overcome by addition of emulsifiers or solvents (such as DMSO or ethanol) which may alter the activity [51]. Antimicrobial action is often determined by more than one component; each of them contributes to the beneficial or adverse effects. The major component may not be the only one responsible for the antimicrobial activity but a synergistic effect may take place with other oil components [50].

According to a literature survey, different mint species have been investigated in search of antimicrobial activities [13,15,48,51,52,53,54], inclusing several analyses performed on MS with an aim to investigate its antibacterial, antifungal or antiviral effects.

Essential oils of MS grown in several regions in Morocco were tested for their activity against 19 bacteria, including Gram-positive and Gram-negative ones, and against three fungi, using solid phase and microtitration assays [23]. The antibacterial and antifungal activities of three types of EOMS were analyzed. Essential oil rich in pulegone strongly inhibited all bacteria, while the activity of that rich in PO was weaker. The activity of the third one (rich in both PO and piperitone oxide) had a tendency to be less important. Those results indicated that the efficacy of essential oils depends on their particular chemical composition, which was confirmed with another analysis where the major aromatic components of those oils were tested against the same organisms‒pulegone was the most active against all bacteria, followed by PO; the activity of piperitone oxide seems to be two-fold lower than that of PO against a number of microorganisms, in particular, yeasts. These results indicate that pulegone may be the most active aromatic component in EOMS [23].

To study the importance of the chemical structure of the major constituents of EOMS, the antimicrobial activity of a series of aromatic components was analyzed and the activities of pulegone, PO and piperitone oxide were compared with carvone, limonene and menthone [55]. These latter aromatic components are also synthesized by *Mentha* species and have less antimicrobial activity. Menthone, which results from the reduction of pulegone, was less active than its precursor. Limonene and carvone also had moderate antimicrobial activity compared with pulegone and PO. Similar results were obtained by other authors [55] who found that pulegone showed a more potent biocidal activity than limonene, carvone and menthone. Compared with pulegone, limonene does not possess the extra cyclic double bond between C4 and C7, which results in the loss of the antimicrobial activity of this compound. Similarly, piperitone oxide does not possess this double bond and had in general a lower activity compared with PO and pulegone. Thus, this double bond seems to be important for the antimicrobial activity of the monoterpenes, possibly by favoring an active configuration of the molecule. The presence of an epoxide between C1 and C2 in PO decreases its antimicrobial activity compared with that of pulegone [23].

Investigation of the aerial parts of MS growing in Egypt showed moderate inhibitory activity against the tested human pathogenic bacteria. In that study, the antimicrobial screening of the ethanolic extract and its subfractions were performed [10]. The oil of the fresh aerial parts showed a potent antifungal activity against *Candida albicans*, *Saccharomyces cerevisiae* and *Aspergillus niger* [43]. Other studies on the Egyptian plant material showed a strong antibacterial activity of the essential oil, especially against *Staphylococcus aureus* [6].

The oils obtained from aerial parts of the wild and cultivated MS ssp. *timija*, an endemic species of Morocco, have been screened for antimicrobial activity. Both oils displayed good to excellent activity against all microorganisms tested, with the oil of the cultivated form being more active [42]. Both essential oils exhibited marked antifungal activity on all the *Candida* species tested, especially against *C. glabrata*. The antimicrobial activities of *timija* mint essential oil can be attributed to the presence of high concentrations of pulegone and menthone, two oxygenated monoterpenes with well-documented antibacterial and antifungal potential [22,37]. However, the comparison of the antimicrobial activity between these two major compounds showed that pulegone has more potent biocidal activity than menthone, which can explain the more potent antimicrobial activity of oil obtained from the cultivated *timija* mint [23,42].

Antimycotic analysis on the oil from the plant material from Tarquinia (Italy) displayed high activity against all strains of *Cryptococcus neoformans* and different dermatophyte strains, such as *Trycophyton mentagrophite*, *T. rubrum*, *T. violacee*, *Microsporum canis* and *M. gypseum*, where in all cases a good antimycotic activity was observed [22]. Another study on that material assessed the *in vitro* and *in vivo* antifungal activity of essential oil in an experimental vaginal candidiasis infection model. That study showed that EOMS was both candidastatic and candidacidal *in vitro*, as demonstrated in an *in vivo* monitoring imaging system [30]. The results obtained from another study demonstrated both the effects of the essential oil on *C. albicans* yeast cells and biofilms, and the synergism of the oil when used in combination with conventional antifungal drugs like fluconazole (FLC) and micafungin (MCFG) [31]. The antifungal activity of this oil was investigated for differences resulting from extraction time and period of material collection. That analysis showed that the oil extracted in the first 3 h showed good antifungal activities, but decreasing activity was evident with the oils from the 6 to 24-hour extractions. The study also confirmed PO as the principal active chemical constituent responsible for the essential oil’s biological activity [47].

Determination of the antibacterial activity of three essential oils was performed *in vitro* against strains of *Pseudomonas syringe* pv. *actinidiae* (PSA), the causal agent of bacterial kiwifruit canker. That study included oil of MS from Tarquinia, which showed significantly higher activity than other two essential oils (*Rosmarinus officinalis* and *Melaleuca alternifolia*). Also, synergistic effects of those three essential oils were analyzed. Treating PSA with the mixture caused a significant decrease in the MIC, compared to their individual values, indicating a significant synergistic effect of the three essential oils combined. Results in that study clearly demonstrated that the mixture was able to kill PSA at the concentration about 16 times lower than the MIC values of the individual oils after 1 h of exposure [56]. This oil was also investigated against *Chlamydia trachomatis*, the most common sexually-transmitted bacterial infection worldwide. Those results showed effectiveness towards *C. trachomatis*, whereby it did not only inactivate infectious elementary bodies but it also inactivated chlamydial replication. The study also revealed the efficacy of the oil in combination with erythromycin: the combination inhibited *C. trachomatis* replication with a considerable four-fold reduction in the minimum effective dose of antibiotic [57].

The effects of EOMS derived from Tarquinia and its active principle PO were also tested in an *in vitro* experimental model of infection with herpes simplex virus type 1 (HSV-1), an important human pathogen. Moreover, a synergistic action was observed in combination with acyclovir. This study demonstrated that the oil, as well as its main compound PO, exerted stronger effects when added post-infection. When the oil, or the oxide, was preincubated with the virus before infection, both showed a significant virucidal activity, thus interfering directly with the viral envelope [32].

The antimicrobial activity of the essential oil from MS growing in the north of Morocco was evaluated on *Salmonella enterica*, *Listeria monocytogenes* and *Escherichia coli*, as well as for antiviral activity on the cytopathogenic murine norovirus (MNV-1). The results showed weak activity against *E. coli* and *L. monocytogenes*, but moderate activity was observed against *S. enterica.* The oil was mainly composed of PO, which was considered to have a low antimicrobial activity due to the presence of an epoxide between C1 and C2. This study showed that the oil tested had low antiviral activity against MNV-1 [39].

The *in vitro* antimicrobial activity of the essential oil of the aerial parts of MS ssp. *insularis* grown in Sardinia was assayed against six *Lactobacillus* species (including four probiotic strains), *Lactococcus lactis* ssp. *lactis* and *Staphylococcus xylosus*. Agar diffusion test results indicated that the essential oil exhibited a low antibacterial activity potential against all tested bacteria, except to *L. lactis* ssp. *lactis* and *S. xylosus* upon which it exerted a slight antibacterial activity. On the other hand, all yeasts strains tested (*Saccharomyces cerevisiae*, *Kloeckera apiculata*, *Candida zemplinina*, *Metschnikowia pulcherrima* and *Tetrapisispora phaffii*) were inhibited, and the oil exhibited slight to strong antifungal activity [44].

The mechanisms by which essential oils inhibit microorganisms involve different modes of action, but may be due in part to their hydrophobicity. As a result, they cause lipid partitioning of bacterial cell membranes and mitochondria, disturbing the cell structures and rendering them more permeable [58,59]. Extensive leakage from bacterial cells or the exit of critical molecules and ions, will lead to death [4]. Impairment of bacterial enzyme systems may also be a potential mechanism of antimicrobial action [60].

The antimicrobial activity of the essential oils can also be explained by the lipophilic character of the monoterpenes contained. The monoterpenes act by disrupting the microbial cytoplasmic membrane, which thus loses its high impermeability for protons and bigger ions. If the membrane integrity is disrupted, then its functions are compromised not only as a barrier but also as a matrix for enzymes and as an energy transducer. However, specific mechanisms involved in the antimicrobial action of monoterpenes remain poorly characterized [31]. According to a number of authors, Gram-negative bacteria are generally less susceptible than Gram-positive bacteria to the actions of essential oils, due to their outer membrane surrounding the cell wall which restricts diffusion of hydrophobic compounds through its polysaccharide covering [61,62,63,64]. According to some authors, this effect seems to be dependent on lipid composition and net surface charge of microbial membranes [62]. This statement is not always true; indeed different authors found no differences or greater sensibility of Gram-negative bacteria than Gram-positive to essential oils [44,52].

## 7. Antioxidant Properties of EOMS

Free radicals are considered to initiate oxidation reactions that lead to aging and cause diseases in human beings. Moreover, activated oxygen incorporates reactive oxygen species (ROS) which consists of free (hydroxyl radicals, superoxide anion radicals) or non-free radicals (peroxide) [65]. These ROS are liberated by virtue of stress, and thus, an imbalance is developed in the body that damages cells in it and causes health problems [18]. On the other hand, restrictions have been imposed on the use of synthetic antioxidants because of their carcinogenicity and other toxic properties, which has considerably increased interest in natural antioxidants [18,19,66].

The active ingredients of a medicinal plant are mainly its secondary metabolites which are naturally produced during the metabolic processes of the plant’s growth [18]. Natural antioxidants can be phenolic compounds (tocopherols, flavonoids and phenolic acids) and carotenoids (lutein, lycopene and carotene) [19,67]. Growing evidence has shown an inverse correlation between the intake of dietary antioxidants and the risk of chronic diseases such as coronary heart disease, cancer and several other aging-related health concerns [68,69]. There are several methods to determine the antioxidant capacity of plant extracts. However, the chemical complexity of extracts could lead to scattered results obtained from different techniques, depending on the test employed. Therefore, an approach with multiple assays in the screening work is highly advisable [70].

Investigation of the antioxidant activity of MS extracts from Morocco showed that the phenol extract presented a high antioxidant activity equivalent to that of butylated hydroxytoluene (BHT), an inhibitor well-known for its antioxidant activity [70,71]. Effectively, phenolic compounds are considered a major group of compounds that contribute to the antioxidant activities of botanical materials because of their scavenging ability on free radicals due to their hydroxyl groups [72]. The antioxidant activity of phenolic compounds is described as being largely influenced by the number of hydroxyl groups on the aromatic ring [73]. This activity is also due to their ability to scavenge free radicals, donate hydrogen atoms or electrons, or chelate metal cations [74]. The highest ferrous ion chelating activity was found in phenol and methanol extracts [71]. There is a linear correlation between the content of total phenolic compounds and their antioxidant capacity [29,72,75,76]. The results of this study indicate that phenolic compounds present in MS could be the major contributors of antioxidant capacities of this species [70].

Nine mint species from Pakistan were investigated as new potential sources of natural antioxidants. The methanolic extract assays revealed that significantly higher activity (82%) was observed in MS [18], which showed appreciable antioxidant activity only in the polar fractions while its decoction was also very effective in the inhibition of AChE and as a scavenger of radicals [28]. *Mentha* species prevent cell damage through their strong antioxidant activity, by scavenging free radicals and neutralizing bacterial invaders. They also promote the release of superoxide dismutase, a powerful antioxidant especially potent in destroying free radicals caused by imbalanced oxidation. Radical scavenging activity was observed when discoloration occurred, and MS were observed to produce high discoloration, followed by the other investigated species [18].

Analysis of some Egyptian mint species gave different results, since the lowest antioxidant activity was found in EOMS—only 6% [6]. Other analysis of the Egyptian EOMS showed potent *in vivo* (96% relative to vitamin E) and moderate *in vitro* antioxidant activities [43]. Ethanolic extracts of aerial parts of MS cultivated in Egypt and its subfractions (*n*-hexane, chloroform, ethyl acetate and *n*-butanol) were also evaluated. The ethyl acetate fraction showed the highest antioxidant activity: *in vivo* (as it restored the glutathione level in diabetic rats by 98%) and *in vitro* as it had the highest free radical scavenging activity (IC_50_ = 31 μg∙mL^−1^) [77]. Extracts of MS gathered from the interior of Portugal only showed appreciable antioxidant activity in the polar fractions [28].

## 8. Insecticidal Activity of EOMS

Plant insecticides have been used to fight pests for centuries. For instance, the use of plant extracts and powdered plant parts as insecticides was widespread during the Roman Empire. However, after the Second World War the few plants and plant extracts that had shown promising effects and were of widespread use were replaced by synthetic chemical insecticides. Later on, the adverse effect of chemical insecticides was realized with the appearance of problems like environmental contamination, residues in food and feed and pest resistance. Since the majority of plant insecticides are biodegradable, this has led to a revival of growing interest in the use of either plant extracts or essential oils. More than 1500 plant species have been reported to have insecticidal value [7]. Many plant secondary metabolites, such as alkaloids, monoterpenoids or phenylpropanoids are toxic to insects; in addition, essential oils extracted from plants have been widely investigated for pest control properties, with some providing to be toxic [78].

*Mentha* has historical significance as a medicinal and insecticidal plant in the traditional knowledge system. In the last few decades, many studies have been reported on the insecticidal activity of several *Mentha* species, largely in terms of adulticidal activity. EOMS from Azrou (Morocco) was investigated for its insecticidal activity against adults of *Sitophilus oryzae*. Considerable differences in insect mortality due to essential oil fumigation were observed using different concentrations and exposure times. Results showed that the essential oil was very toxic against *S. oryzae*, but the degree of this toxicity was influenced by the concentration applied and the exposure time [38]. Another study of the oil from the Moroccan material was carried out on two species of devastating insects of stored foodstuffs: *Sitophilus oryzae* and *Rizopertha dominica*. Mortality was 100% for amounts of 50 µL and 12 µL of the oil, while for the amount of 3 µL an acute toxicity was observed causing the mortality of 85% on the first and 100% on the second day [40]. To assess the biological activity of EOMS (also from Morocco), four concentrations were tested as fumigants against *Callosbruchus maculatus* reared on chickpea seeds. Great effectiveness of the oil was noticed in that study [79]. Oil from the Czech Republic had larvicidal activity against *Culex quinquefasciatus* [45].

The insecticidal activity of essential oils depends closely on their chemical composition. Monoterpenes have been well-documented as active fumigants and insecticides [80] and EOMS contains up to 86.2% of monoterpenes such as PO, pulegone, limonene, piperitenone, β-pinene, α-pinene and *p*-cymene. Their toxicity was proved toward stored product pests [34,40,79,80,81,82,83,84]. The fumigant toxicity of tested oil can be correlated with the abundance of PO. This oxygenated monoterpene possessed high toxicity against pests [45,80,82,84]. Differences in the chemical structures of monoterpenes are another factor influencing biological potency. Oxygenated terpenoids are more toxic than the non-oxygenated ones, and further, even among oxygenated ones, biological activity is differentiated by their other chemical groups and saturation [45]. Other components are present at low levels but may exert a synergistic effect [38].

The mode of action of essential oils and their constituents as insecticides is not known. The lipophilic nature of plant essential oils allows them to interfere with basic metabolic, biochemical, physiological and behavioral functions of insects [7]. Recent studies reported that essential oils and their constituents affect biochemical processes, which specifically disrupt the endocrine balance of insects. They may be neurotoxic or may act as insect growth regulators, disrupting the normal process of morphogenesis [85]. Further, monoterpenes have been investigated for their neurotoxicity [7]; they are typically volatile and rather lipophilic compounds that can penetrate into insects rapidly and interfere with their physiologic functions [86] by inhibiting acetylcholinesterase activity [80,87] and acting on insects’ octopaminergic sites [88].

## 9. Additional Bioactivities of EOMS

EOMS from Egypt was screened for certain other biological activities. It exhibited analgesic and acute anti-inflammatory activities (75% and 82% relative to indomethacin) [43]. In addition, it exerted moderate cytotoxic and hepatoprotective activities [43]. Further investigation included different fractions of the ethanolic extract of the aerial parts of MS growing in Egypt. Analgesic and acute anti-inflammatory activities of the oral administration of the ethanolic extract and its subfractions (*n*-hexane, chloroform, ethyl acetate and *n*-butanol) were evaluated, using indomethacin as a standard drug. As a result, the ethanolic extract showed the most potent analgesic activity (78.5% potency compared to indomethacin), followed by the ethyl acetate and *n*-butanol fractions whose potency percentages were 66.3% and 54.7%, respectively. On the other hand, the ethyl acetate fraction was the most potent anti-inflammatory (88% potency) as compared to indomethacin, followed by the ethanolic extract (82.9%) and *n*-butanol fraction (62.6%) [10]. It is obvious that both analgesic and anti-inflammatory activities were exerted by the ethanol extract, the ethyl acetate and *n*-butanol fractions. It could be concluded that these activities may be attributed to their phenolic contents. The potent anti-inflammatory activities of the ethanolic extract may be due to its content of sterols, triterpenes, phenolic acids and flavonoids which have been proved to exert anti-inflammatory activity [10]. The hepatoprotective activity of the ethanolic extract and its subfractions was also evaluated. It revealed that the ethyl acetate fraction had the highest activity, as it prevented the increase caused by CCl_4_ in the levels of aspartate amino transferase (AST), alanine amino transferase (ALT) and alkaline phosphatase (ALP) enzymes by 51.6%, 57.0% and 56.7%, respectively. The ethanolic extract, as well as its subfractions, were also tested for their cytotoxic activity. The results showed a significant activity of the ethanolic extract on liver carcinoma and larynx cancer cell lines (IC_50_ = 7.28 and 7.35 µg∙mL^−1^, respectively). The ethyl acetate fraction showed the highest activity against human liver carcinoma cell line (IC_50_ = 5.1 µg∙mL^−1^), the chloroform fraction was the most potent on the colon carcinoma cell line (IC_50_ = 14.40 µg∙mL^−1^) while *n*-hexane was the most active regarding the breast carcinoma cell line (IC_50_ = 13.5 µg∙mL^−1^) [77].

Essential oils, ethanolic extracts and decoction of 10 plants from interior Portugal were analyzed for their activity towards acetylcholinesterase (AChE) enzyme. MS showed AChE inhibitory capacity higher than 50% in the essential oil fraction, while the ethanolic extract was less potent. A high value of AChE inhibitory activity was found in a decoction of MS [28].

The pharmacological activity of a methanol extract of the leaves and stems of MS was analyzed in *in vivo* and *in vitro* models. The extract exhibited a central nervous system depressant action but no anticonvulsive activity. In order to determine the type of analgesia induced, the activity was evaluated using three different pain stimuli, *i.e.*, heat, mechanical and chemical agents such as acetic acid. The extract evaluated lacked any analgesic effect arising from CNS action since it did not increase the response time in the hot plate test. However, the extract showed a significant effect on mechanical and chemical stimuli, thus suggesting the induction of a peripheral analgesic effect. The extract also showed significant anti-inflammatory action inhibiting the rat paw oedema induced by carrageenan. Moreover, the *in vitro* studies showed a significant diminution in the contractile effects induced by histamine, serotonin and acetylcholine [14].

Since MS preparations have been applied in the traditional medicine of the Mediterranean areas as a hypotensive, the methanol and dichloromethanol extracts of the leaves and stems of this plant were tested for their effects on resting arterial blood pressure, heart rate and noradrenaline induced hypertension. Both extracts reduced the mean arterial blood pressure and heart rate, while only the dichloromethanol extract prevented the noradrenaline induced hypertension [27]. Mutagenicity tests revealed that the oil was not endowed with any particular toxic effect [47].

## 10. Piperitenone Oxide—the Main EOMS Chemical Components

**Scheme 2:**
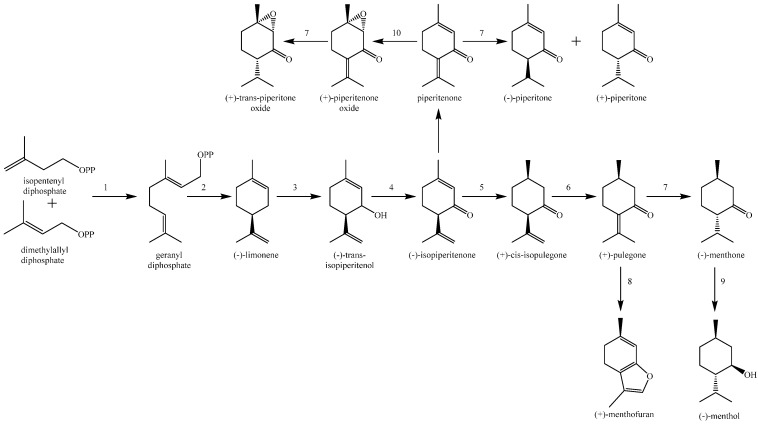
The principal pathways for monoterpene biosynthesis in peppermint. The responsible enzymes are as follows: geranyl diphosphate synthase (**1**); (−)-limonene synthase (**2**); cytochrome P450 (−)-limonene-3-hydroxylase (**3**); (−)-trans-isopiperitenol dehydrogenase (**4**); (−)-isopiperitenone reductase (**5**); (+)-cis-isopulegone isomerase (**6**); (+)-PR (**7**); cytochrome P450 (+)-MFS (**8**); (−)-menthone reductase (**9**); and the terpenoid epoxidase (**10**). OPP denotes the diphosphate moiety [94].

1,2-Epoxypulegone (PO) is an important chemical constituent of the essential oils of many *Mentha* species, where it is formed by epoxidation of piperitenone (Scheme 2). It was firstly named rotundifolone, since it was isolated from the essential oil of *M. rotundifolia* cultivated in Japan [89,90]. The taxonomic status of this species is confusing. Namely, according to Flora Europea [8], this was a misapplied name for MS (*Mentha rotundifolia* auct., non (L.) Huds.) and some authors consider it as its synonym [91], as mentioned in some articles [33,35,92]. On the other hand, there is a hybrid of MS and *M. longifolia*. named *Mentha* × *rotundifolia*. This problem has been discussed and clarified by some authors [93].

This monoterpenoid ketone (C_10_H_14_O_2_, molecular weight 166) is highly dextro-rotatory and has a low melting point (27.5 °C). The wavelengths of the ultraviolet absorption of PO and its semicarbazone are 260 and 273 nm, respectively, which indicates that this oxide has an α,β-unsaturated carbonyl system. PO does not give a ferric chloride reaction in methanol nor in aqueous suspension, but shows a positive Malaprade reaction. It reduces ammoniacal silver nitrate and Fehling solution. PO is soluble in ethanol, methanol, benzene and petroleum ether, but not in water and aqueous alkali [91]. PO is considered as one of the major common constituents of EOMS. This oxygenated monoterpene exhibits interesting activities, including cardiovascular, antimicrobial and insecticidal [38,79,82].

## 11. Alternative Sources of PO

Some other mint species are also rich in PO, which is often the major constituent of the essential oil of *Mentha x villosa*. The percentage varies from 55.4 to even 98% [95,96,97,98,99,100,101,102,103]. The oil of *M. microphylla* contains also PO in amounts of 32.9% [104] or even 65% [105]. Different investigations indicate high level of PO in the oils of *M. spicata* (up to 94.8%) [45,106,107,108,109,110,111] and *M. rotundifolia* (80.8%) [33,35,112], respectively. The last one was actually *Mentha* × *rotundifolia* (the hybrid) as explained by authors themselves. The analysis of the *M. longifolia* oils also showed a significant amount of PO: ranging from 14.7% to 83.7% [12,19,45,52,113,114,115,116]. *M. aquatica* var. *crispa* oil from South Vietnam is also recognized as a rich source of PO (up to 91.2%) [117].

According to recent investigations, the plant species *Lippia pedunculosa* (Verbenaceae) may be considered as the new important source of PO since it is a predominant constituent (≈72%) in its essential oil [118]. For sure, PO is not characteristic of the genus *Lippia*, but some of the other species also contains it as a minor or significant constituent [119]. Thus, *L. turbinate* essential oil contains 30% [120], the amount in *L. juneliana* oil is from 22.9% to 47.7% [121], while the oil of *L. alnifolia* was shown to have 44.6% of PO [122].

PO is also found to be the main constituent of some *Satureja* oils (Lamiaceae). Thus, *S. parvifolia* from Argentina contains 69.8% of PO [123,124], while the endemic *S. kallarica* from Iran has a 71.2% content [125]. Some *Calamintha* species (Lamiaceae) are also recognized to be quite rich in PO. The oils of *C. incana* from Turkey [126] and *C. nepeta* ssp. *glandulosa* from Belgium [127,128] contain up to 66.6% and 52% of PO, respectively.

**Scheme 3:**
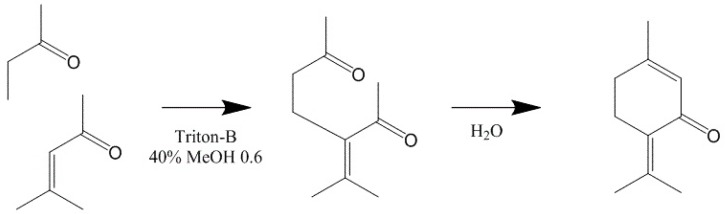
Synthesis of piperitenone from mesityloxide and methyl vinyl ketone.

Besides being available from natural sources, PO can be also produced by chemical synthesis. One of the ways is the epoxidation of piperitenone [119]. The monoterpenoid piperitenone (**1**) is of interest because it can be converted to a wide variety of important compounds. It has been obtained by several chemical routes [129,130]. The best yield was achieved with the process involving condensation of mesityl oxide (**2**) in tetrahydrofuran (THF) with methyl vinyl ketone (**3**) in THF and a solution of Triton-B (Scheme 3) [131].

Another synthetic procedure involves the same condensation, but uses sodium *tert*-butoxide as condensing agent in toluene [129]. However, this process mainly yields isoxylitone, the self-condensation product of **2** and less than 8% of **1**. Subsequently, numerous investigations have attempted to attain a high yield of **1** by suppressing simultaneously the formation of isoxylitone. It had been found that when an alkali metal alkoxide was used as condensing agent, the self-condensation of **2** occurred in an aprotic solvent (e.g., *n*-hexane, benzene) but little or no reaction occurred if the solvent was replaced by a protic solvent (e.g., ethanol), tetrahydrofuran (THF) or a hydrous aprotic solvent. A heterogeneous system of potassium hydroxide and THF restricts the formation of isoxylitone considerably [130]. It has been reported that **1** is capable of forming a water-soluble sodium bisulfite addition compound. Treatment of the reaction mixture from the condensation reaction with bisulfite followed by ether extraction gave an aqueous solution from which **1** could be regenerated and isolated in 54% overall yield. Another condensation includes sodium hydride or a potassium 2-butoxide as catalysts [130].

**Scheme 4:**
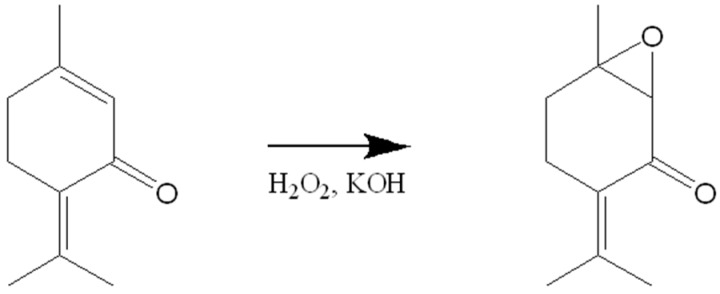
Epoxidation of piperitenone.

In the epoxidation step 30% aqueous hydrogen peroxide and 10% potassium hydroxide should be added to **1** in isopropyl alcohol. Two main fractions are obtained: the unreacted **1** mixed with PO, which could be separated by the column chromatography (Scheme 4) [119].

## 12. Bioactivities of PO

The monoterpenic ketone PO has been evaluated in relation to the following different biological activities: cardiovascular, hypotensive, bradycardic, insecticidal, trypanocidal, schistosomicidal, antimicrobial and antinociceptive properties.

It has been shown that PO exhibited central analgesic activity in mice and rats [97]. PO was analyzed for potential activity on smooth muscle. The experimental model was the guinea pig ileum, and the main conclusion of that study was that PO had a relaxant, depressant activity on intestinal smooth muscle [132].

PO exhibited strong toxic effect against the larvae of the mosquito species *Aedes aegypti* [103] but also against other mosquito species [82,84]. Although it has shown a good larvicidal effect against the *Ae. aegypti*, it was less potent than its essential oil of origin, which may be due to a synergistic interaction between PO and other compounds. Comparing PO and its analogues confirmed that the different functional groups and their positions in the *p*-menthane skeleton influence the larvicidal activity. In general, replacement of C=C double bonds by epoxide groups decreases the larvicidal potency. It can be concluded that with appropriate structural modification in the monoterpenes it may be possible to develop new larvicidal agents [103].

The essential oil from the leaves of *Lippia pedunculosa* and its main compounds, the monoterpenes PO and (*R*)-limonene, were evaluated for their trypanocidal activity against epimastigote and trypomastigote forms of *Trypanosoma cruzi.* PO was the most active compound. The effects of the oil and isolated compounds on the intracellular form of the parasite were also evaluated in cultures of macrophages infected with *T. cruzi*, but the treatment with (*R*)-limonene and PO caused a moderate reduction in the percentage of macrophages [118].

Biological effects of the *M. x villosa* essential oil and its main constituent PO were evaluated on adult worms of *Schistosoma mansoni* (Plathelminths). Worms were incubated with different concentrations of the oil and PO, which resulted in decreased worm motility continuing until 96 h of observation. At higher concentrations (100 and 70.96 µg∙mL^−1^, respectively), both the essential oil and PO caused mortality among adult *S. mansoni* worms [101].

Cardiovascular effects of intravenous treatment with the essential oil of *M. x villosa* were investigated. Additionally, that study examined whether the major constituent PO was the active principle mediating changes in mean aortic pressure and heart rate. The study showed that the treatment with oil in rats induced hypotensive and bradycardic effects, which appeared mostly attributed to the action of the main compound of the oil, PO [95]. The acute cardiovascular effects of PO were also investigated in rats by using a combined (*in vivo* and *in vitro*) approach. The acute administration of PO induced a short-lasting and dose-related decrease in arterial pressure, followed by a significant bradycardia, probably due to a non-specific muscarinic receptor stimulation. Furthermore, *in vitro* studies suggested that PO induced vasodilatation [97]. The effects of PO on vascular smooth muscle were analyzed, and the major finding was that PO-induced vasodilatation of the rat aorta was apparently mediated by an inhibitory effect on Ca^2+^ influx and inhibition of intracellular Ca^2+^ release from stores [96]. The authors concluded that the hypotensive effect was possibly due to a reduction in heart rate associated to a reduction of peripheral vascular resistance, both due to muscarinic activation [133]. PO induces muscle contraction in both depolarized and non-depolarized sartorius muscle of toad (*Bufo paracnemis*). The study demonstrated that PO induced muscle contraction by releasing calcium from the sarcoplasmic reticulum, probably by the activation of the ryanodine receptor [134].

Assessment of the antinociceptive activity of PO and the analogous compounds was performed using the acetic acid-induced writhing model in mice. All compounds showed to be more antinociceptive than PO against the pain response induced by acetic acid. It was found that the functional groups and their position on the ring of PO contributed to its antinociceptive activity [135]. All those hypotheses were subsequently strengthened by studies characterizing the molecular mechanism of action involved in relaxation produced by PO. The findings suggested that PO induced vasodilatation through two distinct but complementary mechanisms that clearly depended on the concentration used [136]. Another study was performed to evaluate the vasorelaxant effects of different monoterpenes and establish the structure-activity relationship of PO and its structural analogues. The results showed that both oxygenated and non-oxygenated monoterpenes exhibited relaxation activity. The absence of an oxygenated molecular structure was not a critical requirement for the molecule to be bioactive. It was also found that the position of ketone and epoxide groups in the monoterpene structures influenced the vasorelaxant potency and efficacy [137].

The oil of *M. x villosa* and its major component PO together with four similar analogues (limonene oxide, pulegone oxide, carvone epoxide and (+)-pulegone) were evaluated in relation to the antimicrobial activity against *Staphylococcus aureus*, *Escherichia coli*, *Pseudomonas aeruginosa*, *Candida albicans* and a strain of meticilin-resistant *Staphylococcus aureus* (MRSA). The essential oil, PO and the analogues showed antibacterial activity on *S. aureus* and antifungal activity on *C. albicans*. Limonene oxide and carvone epoxide were the substances with the lowest antimicrobial potential. None of the products showed antimicrobial activity against strains of the Gram negative bacteria *E. coli* and *P. aeruginosa* [138].

## 13. Conclusions and Future Perspectives

In general, plants have provided a source of inspiration for novel drug compounds. The increased interest in alternative natural substances is driving the research community to find new uses and applications for these substances and has led to a considerable increase in the use of medicinal plants.

The results of the cited studies indicate that MS and PO, as the main compound of EOMS, show a wide range of biological activities. Keeping in mind that the biological properties can be the result of synergism, investigation of the main compounds alone seems questionable. However, PO is usually the predominant compound (sometimes more than 90%) in EOMS and was found to reflect quite well the biophysical and biological features of the whole oil. PO seems to be responsible for a lot of bioactivities although it is possible that its activity is slightly modulated by the other minor molecules.

Neither the essential oil nor PO have very strong antibacterial effects. Pulegone is the most important *Mentha* constituent responsible for antibacterial activity, but the oil of this species is usually not rich in it. The effect of the oil is significant only in cases where other compounds are present in reasonable amounts, such as pulegone, menthone or β-cymene. On the other hand, the oil and PO seem to be potent antifungal agents. In that sense, further investigations of PO can be proposed. There are some data indicating certain antiviral effects of the oil, as well as PO. Thus, that field is also interesting for the future examinations. It should be added that the further studies are needed to evaluate the *in vivo* potential in animal experimental models since there are little data about that aspect.

The essential oil has strong antioxidant potential. In some cases, it can be compared with butylated hydroxytoluene which is a well-known synthetic antioxidant additive. However, to the best of our knowledge, investigation of PO in this field is missing.

Insecticidal properties of EOMS and PO were evaluated. Since they usually exhibit strong toxic effects, this can definitely be a field for future study. The oil contains a huge amount of monoterpenes (often, even more than 85%) which have been well-documented as active fumigants and insecticides. On the other hand, oxygenated terpenoids are more toxic than the non-oxygenated ones. Thus, the fumigant toxicity of this oil can be justified by the high content of the monoterpene PO, which has already been well explored in that sense. What can be emphasized is that appropriate structural modification in the monoterpenes may lead to the development of new insecticidal agents.

Additional bioactivities of EOMS, as well as PO, have been investigated, and the results showed good potential in relation to analgesic, anti-inflammatory, antihypertensive and AChE inhibitory activities. There are also a significant number of studies on the different extracts, not the essential oil, that can be continued avenues of study in the future. Future studies should further explore the possible beneficial synergistic properties of combining PO (or the whole oil) with other natural or synthetic compounds.
